# The Impact of Empathy and Demographic Factors on First-Year Health Students’ Perceptions of an Age-Friendly Campus

**DOI:** 10.1093/geroni/igaf122.2508

**Published:** 2025-12-31

**Authors:** Michal Elboim-Gabyzon, Sigal Pearl Naim, Malka Doron, Tal Kohli-Hailovski, Anna Zisberg

**Affiliations:** University of Haifa, Haifa, HaZafon, Israel; University of Haifa, Haifa, HaZafon, Israel; The Center for Research and Study of Aging, University of Haifa, Israel; University of Haifa, Haifa, HaZafon, Israel; University of Haifa, Haifa, HaZafon, Israel

## Abstract

The concept of an “Age-Friendly-University” embodies a paradigm that fosters inclusivity and intergenerational engagement in higher education. As populations age, universities should support both traditional students and older adults, cultivating professional attitudes that value contributions from all age groups. To facilitate this transformation, it’s essential to assess students’ perceptions regarding age-inclusivity. We hypothesized that demographic factors and empathy influence students’ these perceptions, grounded in emotional intelligence and intergroup contact theories, which suggest that higher empathy decreases ageist biases and promotes intergenerational connections. This study examined factors shaping first-year health students’ views on age inclusivity and campus age-friendliness using subscales of the Age-Friendly Inventory and Campus Climate Survey (ICCS) and whether empathy place a role. A cross-sectional survey was administered to 248 health students (mean age = 23.87±4). Regression analyses of demographic characteristics including age, volunteering, working with older adults and ageistic-attitudes, revealed that age was the only significant predictor for Personal Beliefs about Age Inclusivity (β = 0.193, p = 0.003). Including the Interpersonal Reactivity index as a measure of empathy identified it as a central predictor (β = 0.315, p < 0.001), almost dabbling the explained variance. Perceptions of campus age-friendliness were significantly predicted by better economic status (β = 0.109, p = 0.026) and lower ageist attitudes (β = 0.152, p < 0.01). These findings underscore empathy’s vital role in shaping age-inclusive attitudes. Developing structured, experiential learning opportunities that enhance students’ empathy may serve as a transformative strategy for embedding age-friendly values within healthcare education, preparing future professionals for intergenerational settings.

